# Supplementary material for: Blood-based detection of MMP11 as a marker of prostate cancer progression regulated by the ALDH1A1-TGF-β1 signaling mechanism
Source: J Exp Clin Cancer Res. 2025 Mar 24;44:105. doi: 10.1186/s13046-025-03299-6 (PMC11931756; doi:10.1186/s13046-025-03299-6)

Supplementary information

Table S1. Primers, siRNA oligonucleotides, shRNAs and antibodies used for the study.

siRNA oligonucleotides used for knockdown of gene expression

Gene	siRNA name	Sequence
ALDH1A1	siALDH1A1	ss UCACAUGGAUAUAGACAAAdTdT<br>as UUUGUCUAUAUCCAUGUGAdTdT
		ss GAUCCAGGGCCGUACAAUAdTdT<br>as UAUUGUACGGCCCUGGAUCdTdT
ALDH1A3	siALDH1A3	ss AGGAAAUGGCAGAGAACUAdTdT<br>as UAGUUCUCUGCCAUUUCCUdTdT
		ss UCGUGGAGGAGCAGGUCUAdTdT<br>as UAGACCUGCUCCUCCACGAdTdT
Scrambled	siSCR	ss GCAGCUAUAUGAAUGUUGUdTdT<br>as ACAACAUUCAUAUAGCUGCdTdT
		ss UGCGCUAGGCCUCGGUUGCdTdT<br>as GCAACCGAGGCCUAGCGCAdTdT
AR	siAR	ss CCAAAGGGCUAGAAGGCGAdTdT<br>as UCGCCUUCUAGCCCUUUGGdTdT
		ss AUUGAUAAAUUCCGAAGGAdTdT<br>as UCCUUCGGAAUUUAUCAAUdTdT
RARA	siRARA	ss CUGUGAGAAACGACCGAAAdTdT<br>as UUUCGGUCGUUUCUCACAGdTdT
		ss CUGCGAAGCAUCAGCGCCAdTdT<br>as UGGCGCUGAUGCUUCGCAGdTdT
		ss UAAAGGUCUACGUGCGGAAdTdT<br>as UUCCGCACGUAGACCUUUAdTdT
		ss CGGAUCUGCACGCGGUACAdTdT<br>as UGUACCGCGUGCAGAUCCGdTdT
RXRA	siRXRA	ss GGCAAGGACCGGAACGAGAdTdT<br>as UCUCGUUCCGGUCCUUGCCdTdT
		ss CGAACGACCCUGUCACCAAdTdT<br>as UUGGUGACAGGGUCGUUCGdTdT
		ss UGACGGAGCUUGUGUCCAAdTdT<br>as UUGGACACAAGCUCCGUCAdTdT
		ss CAGCCGGGAAGGUUCGCUAdTdT<br>as UAGCGAACCUUCCCGGCUGdTdT
MMP11	siMMP11	ss CCUACAGGAUCCUUCGGUUdTdT<br>as AACCGAAGGAUCCUGUAGGdTdT
		ss GUGCUGACAUCAUGAUCGAdTdT<br>as UCGAUCAUGAUGUCAGCACdTdT
		ss GGGCGUUCAACACCUAUAUdTdT<br>as AUAUAGGUGUUGAACGCCCdTdT
		ss GGGAUAGACACCAAUGAGAdTdT<br>as UCUCAUUGGUGUCUAUCCCdTdT

Primers used for qPCR

Gene	Sequence (5’→3’)
ACTB	F 5'- ATGGAGTCCTGTGGCATCCA-3'<br>R 5'- AGTACTTGCGCTCAGGAGGA-3'
RPLP0	F 5'- CTCAACATCTCCCCCTTCTCCTT-3'<br>R 5'- TGATGCAACAGTTGGGTAGCC-3'
ALDH1A1	F 5'- GAATGGCATGATTCAGTGAGTGG-3'<br>R 5'- CAGCCAACTTGTATAATAGTCG-3'

ALDH1A3	F 5'- TCTCGACAAAGCCCTGAAG-3'<br>R 5'- TATTCGGCCAAAGCGTATTC-3'
AR	F 5'- CATCTTGTCGTCTTCGGAAATGTTA-3'<br>R 5'- GAAGCCTCTCCTTCCTCCTGTAGTT-3'
RARA	F 5'- CAAGTGCATCATTAAGACTGTGG-3'<br>R 5'- CGAGAAGGTCATGGTGTCC-3'
RXRA	F 5'- GCTGCACGTCCACCGGAAC-3'<br>R 5'- CCTTGGAGTCAGGGTTAAAGAGG-3'
MMP11	F 5'- GGACCTCACCTACAGGATC-3'<br>R 5'- GTCCCCATGCCAGTACCTG-3'
KLK3	F 5'- GTCTTCCTCACCCTGTCCGTGAC-3'<br>R 5'- GCAGTGGGCAGCTGTGAGGAC-3'
SMAD3	F 5'- GCGTGCGGCTCTACTACATC-3'<br>R 5'- GCACATTCGGGTCAACTGGTA-3'
TGFB1	F 5'- CAGCAGGGATAACACACTGC-3'<br>R 5'- CACGCAGCAGTTCTTCTCC-3'
Aldh1a1 (*Mus musculus*)	F 5'- ATACTTGTCGGATTTAGGAGGCT-3'<br>R 5'- GGGCCTATCTTCCAAATGAACA-3'
Aldh1a3 (*Mus musculus*)	F 5'- GGGTCACACTGGAGCTAGGA-3'<br>R 5'- CTGGCCTCTTCTTGGCGAA-3'
Mmp11 (*Mus musculus*)	F 5'- CTGCCTCTGCTGCTCCTGTTG-3'<br>R 5'- GCAGGACTAGGGACCCAATG-3'
Actb (*Mus musculus*)	F 5'- CCCTAGGCACCAGGGTGTG-3'<br>R 5'- CACGGTTGGCCTTAGGGTTC-3'
Gapdh (*Rat / mouse*)	F 5'- TTCAACGGCACAGTCAAGG-3'<br>R 5'- ACATACTCAGCACCAGCATCAC-3'

Primers used for ChIP-qPCR

**Gene**	**Sequence (5'→3')**
prKLK3	F 5'- GCAAAGGATCTAGGCACGTGAG-3'<br>R 5'- CACCCAGAGCTGTGGAAGG-3'
prRIG-1	F 5'- GCAGCTGTACTTATACCCAC-3'<br>R 5'- CTAGCAGAGGGTGATGGAAG-3'
prTGFB1 #1	F 5'- GCAGTTGGCGAGAACAGTTGG-3'<br>R 5'- GCTCTTGACCACTGTGCCATCC-3'
prTGFB1 #2	F 5'- GGATGGCACAGTGGTCAAGAGC-3'<br>R 5'- CACCTGTAAGAATTGCTCTCC-3'

Antibodies used for ChIP

**Antibodies**	**Vendor and catalogue number**
AR<br>RARA<br>IgG	Cell Signaling Technology, #5153<br>Cell Signaling Technology, #62294<br>Cell Signaling Technology, #3900

shRNA constructs used for knockdown of Mmp11

**Gene**	**Name**	**Vector**	**Hairpin sequence**
Non-silencing	shNS	pLKO.1	CCGGTCCTAAGGTTAAGTCGCCCTCGCTCGA<br>GCGAGGGCGACTTAACCTTAGGTTTTTG
Mmp11	shMmp11 #1	pLKO.1	CCGGCTATGATGAAACTTGGACTATCTCG<br>AGATAGTCCAAGTTTCATCATAGTTTTTG
Mmp11	shMmp11 #2	pLKO.1	CCGGGACCTGCCTGATGTACTGAATCTCGAG<br>ATTCAGTACATCAGGCAGGTCTTTTTG

Table S2. Gene sets used for the correlative analysis. The gene lists are retrieved from the commercially available RT2 Profiler Arrays (Qiagen).

Androgen Receptor Signaling Targets: SORD PGC ERRFI1 TIPARP FZD5 IGF1R GUCY1A3 FOS
HERC3 ZNF189 NKX3-1 NFKB2 HPGD DBI NFKBIA CITED2 APPBP2 CYP2U1 ABHD2 MAF
MYC SNAI2 ADAMTS1 SP1 ORM2 ORM1 ELL2 KLK3 KLK2 RAB4A ELK1
PAK1IP1 SLC26A2 SPDEF SMS CENPN REL SEC22C SGK1 ACKR3 NDRG1 TRIB1 KLK4
IGFBP5 TSC22D1 DHCR24 NFKB1 RELA VAPA MME PIAS1 SRF PIK3R3 LRIG1
LRRFIP2 RHOU EAF2 WIPI1 ENDOD1 STEAP4 ZBTB10 ABCC4 PMEPA1 MT2A SLC45A3 LIFR
STK39 IRS2 NCAPD3 FAM105A TMPRSS2 ACSL3 MAP7D1 JUN CAMKK2 VIPR1 PPAP2A
ZBTB16 TPD52 TSC22D3 AR ALDH1A3 FKBP5 KRT8

Extracellular Matrix and Adhesion Molecules: SPP1 MMP12 MMP7 VTN MMP10 MMP14 MMP13 ITGB5
HAS1 SELP MMP15 ECM1 SELL MMP1 SELE COL12A1 TIMP2 VCAM1 SPARC
ADAMTS13 MMP11 TNC ITGAV ITGAL CTGF COL6A2 ADAMTS1 THBS1 LAMC1
THBS3 ITGB2 COL6A1 TIMP3 COL7A1 SGCE TGFBI ITGA2 MMP3 MMP9 CTNNA1
CNTN1 COL5A1 MMP8 ITGA5 CLEC3B MMP2 THBS2 COL16A1 ITGA4 CDH1 LAMA3
COL4A2 ICAM1 LAMA1 ITGA3 CD44 CTNND1 FN1 COL1A1 ITGB4 NCAM1 LAMA2 TIMP1
ITGB1 CTNNB1 ADAMTS8 LAMB1 ITGA7 COL11A1 VCAN ITGA6 COL15A1 ITGAM SPG7
LAMB3 CTNND2 COL14A1 ITGA1 ITGA8 COL8A1 ITGB3 PECAM1 MMP16 KAL1

Epithelial to Mesenchymal Transition: SPP1 TGFB1 BMP1 FGFBP1 TGFB3 ILK SMAD2 ZEB2 SNAI1
STEAP1 TWIST1 BMP2 RGS2 IL1RN GNG11 MSN DSC2 WNT11 SPARC TMEM132A
ERBB3 ZEB1 GSC ITGAV BMP7 MST1R TCF3 TCF4 SNAI2 COL5A2 ESR1
TGFB2 MMP3 MAP1B MMP9 OCLN FOXC2 PDGFRB CAV2 AKT1 KRT14 ITGA5 JAG1
MMP2 TFPI2 GEMIN2 CDH1 COL3A1 PTK2 CAMK2N1 VIM FN1 KRT19 GSK3B DESI1
STAT3 CDH2 TIMP1 SNAI3 ITGB1 SERPINE1 CTNNB1 NODAL PLEK2 AHNAK VCAN
IGFBP4 TMEFF1 TSPAN13 NOTCH1 RAC1 SOX10 WNT5A NUDT13 COL1A2 CALD1 FZD7
WNT5B DSP EGFR PTP4A1 F11R VPS13A KRT7

Angiogenesis: CXCL1 IFNG TGFB1 ANPEP FGFR3 MMP14 CXCL8 ANGPTL4 FIGF IFNA1 HPSE
CCL11 F3 SPHK1 VEGFA CDH5 ENG CXCL9 VEGFB TIE1 PF4 MDK TEK
CXCL5 TIMP2 IL1B NRP1 EFNB2 ID1 CXCL10 ITGAV PLG S1PR1 CTGF FLT1
KDR PDGFA IL6 HIF1A THBS1 COL18A1 PLAU CXCL6 PGF TNF VEGFC
SERPINF1 TIMP3 TGFB2 MMP9 EFNA1 AKT1 JAG1 MMP2 THBS2 EPHB4 IGF1 EGF
HGF EDN1 LECT1 FN1 CCL2 TGFA TIMP1 NRP2 NOTCH4 SERPINE1 ADGRB1 FGF2
PROK2 TGFBR1 COL4A3 ANG NOS3 PTGS1 TYMP ERBB2 LEP ANGPT1 ANGPT2 FGF1
ITGB3 PECAM1

Cell Motility: MAPK1 TGFB1 MMP14 ILK RND3 MYH10 PAK4 IGF1R CFL1 LIMK1
VEGFA CAV1 WASF1 PIK3CA MSN TIMP2 WIPF1 BAIAP2 MET WASL CDC42 DPP4
CSF1 RHOA RHO ITGB2 PLAUR SRC RHOC RHOB RAC2 PFN1 CAPN2 MYH9
BCAR1 MMP9 VASP ARHGDIA AKT1 MMP2 ROCK1 PXN PTK2B ITGA4 EZR
CAPN1 IGF1 ACTN1 ARF6 MYL9 EGF HGF WASF2 PTK2 VIM PTPN1
STAT3 PLCG1 RASA1 PLD1 ENAH PTEN ACTN4 ARHGEF7 ITGB1 CTTN SVIL
ACTR2 FAP MYLK FGF2 DIAPH1 TLN1 PRKCA RDX VCL RAC1 CRK
SH3PXD2A EGFR ITGB3 PAK1 ACTR3 ACTN3

Osteogenesis: SPP1 BGN TGFB1 BMP1 CSF2 TGFB3 GDF10 MMP10 SOX9 COMP CSF3
SMAD2 IGF1R SMAD3 TWIST1 BMP4 VEGFA BMP2 ALPL SMAD4 VEGFB TGFBR2 DLX5
VCAM1 CDH11 CD36 BMP3 RUNX2 ACVR1 BMP7 CSF1 ANXA5 COL2A1 IHH FLT1
PDGFA PHEX SERPINH1 TNF ITGA2 TGFB2 MMP9 CALCR BMPR2 FGFR2 CHRD IGF2
COL5A1 MMP8 MMP2 BMPR1B NOG ICAM1 COL3A1 IGF1 EGF ITGA3 NFKB1 BMP5
FN1 COL1A1 CTSK FGFR1 VDR ITGB1 BMP6 FGF2 TGFBR1 AHSG COL10A1
SMAD1 COL15A1 ITGAM SP7 SMAD5 COL1A2 COL14A1 ITGA1 GLI1 EGFR FGF1
TNFSF11 BMPR1A BGLAP

Tumor Metastasis: CXCR2 TGFB1 MMP7 MMP10 MMP13 NME4 SMAD2 DENR HRAS HPSE
VEGFA SMAD4 GNRH1 IL18 HTATIP2 KISS1 BRMS1 TIMP2 MGAT5 CDH11 NME1 IL1B
MET MMP11 TSHR CHD4 MYC CCL7 CD82 MYCL FLT4 FXYD5 TP53 CTSL
TNFSF10 PLAUR CXCR4 SRC ETV4 PNN TIMP3 MMP3 RPSA MMP9 CTNNA1
CDKN2A MDM2 CTBP1 CST7 MMP2 APC TRPM1 CDH1 COL4A2 IGF1 CD44
NR4A3 MCAM HGF RORB FN1 MTA1 CTSK SSTR2 PTEN KISS1R SERPINE1
METAP2 KRAS SYK ITGA7 EWSR1 TCF20 MTSS1 TIMP4 EPHB2 CXCL12 RB1
FGFR4 NF2 SET ITGB3 CDH6 FAT1

TGFb Signaling Targets: ATF3 RYBP AGT FOS SMAD3 SNAI1 VEGFA ENG ID3
NFKBIA RAD21 TGFBR2 MSX2 PTHLH PPARA E2F4 GADD45B SHH SMAD6 BACH1 ID1
IFRD1 ACVR1 CRYAB PLG ATF4 MYC RHOA BDNF PDGFA BRD2 RUNX1
THBS1 SP1 MYOD1 TNFSF10 KLF10 MBD1 RHOB CDKN1B S100A8 IL10 FURIN
TGFB2 BHLHE40 HEY1 HERPUD1 MMP2 CDC6 MAPK8 PTK2B DNAJA1 ID2 CEBPB
HMOX1 TXNIP BCL2L1 NFIB PTK2 FN1 MAP3K7 SERPINE1 HES1 CREB1 CTNNB1 AIPL1
RARA EMP1 SMAD1 RBL1 EPHB2 GTF2I GLI2 EP300 NOTCH1 CREBBP SMAD5
ACVRL1 MAPK14 PTGS2 ACTA2 AR SREBF2 SOX4

TGFb BMP Signaling Pathway: DLX2 TGFB1 BMP1 GDF5 INHBB PDGFB TGFB3 FST
SMAD2 JUNB FOS INHBA SMAD3 LTBP1 BMP4 CDKN2B BMP2 SMAD4 ENG
TGFBR2 GDF3 GADD45B AMH BMP3 ID1 IFRD1 ACVR1 GSC BMP7 ATF4 MYC
IL6 INHA RUNX1 THBS1 TNFSF10 PLAU CDKN1B TGFB1I1 LTBP2 TGFBI TGFB2
BMPR2 CHRD HIPK2 HERPUD1 ID2 IGFBP3 BMPR1B NOG IGF1 TSC22D1 TGFBR3 BMP5
SMURF1 COL1A1 AMHR2 ACVR2A STAT1 SERPINE1 BMP6 SMAD7 NODAL LEFTY1 TGFBR1 EMP1
SMAD1 TGFBRAP1 SMAD5 ACVRL1 JUN BAMBI COL1A2 GDF6 GDF2 BMPER
CDKN1A BMPR1A SOX4 TGIF1 MECOM GDF7 BGLAP DCN

Supplementary Figures

Figure S1. (**A**) Analysis of the ALDH1A1 and ALDH1A3 knockdown. qPCR analysis of the relative ALDH1A1 and ALDH1A3 expression in LNCaP, C42B and PC3 cells upon knockdown of ALDH1A1 or ALDH1A3. Cells transfected with scrambled siRNA (siScr) were used as controls. n = 3; Error bars = SD; **p < 0.01; ***p < 0.001. (**B**) The Kaplan-Meier analyses of the biochemical recurrence-free survival (BRFS) and metastasis-free survival (MFS) for patients with high-risk and locally advanced PCa treated with ADT and then concurrently with one of three radiotherapy regimens as described in Table 3. The patients were stratified by the most significant cut-off for ALDH1A3 expression levels. (**C**) Correlation of mRNA expression levels of ALDH1A1 and ALDH1A3 genes and gene sets related to osteogenesis, angiogenesis, extracellular matrix (ECM) and adhesion in the TCGA PRAD patient cohort (n = 490). The gene lists are provided in Table S2. PCC - Pearson correlation coefficient. **D**) Correlation of mRNA expression levels of ALDH1A1 and ALDH1A3 genes and gene sets related to tumor metastasis in the SU2C patient cohort (n = 266) and MSKCC patient cohort (n = 185). The gene list is provided in Table S2. SRCC - Spearman's Rank correlation coefficient.

Figure S2. (**A**) qPCR analysis of RARA expression in LNCaP cells upon transient RARA overexpression, treatment with ATRA or 9-cis retinoic acid (9CisRA) for 48 h or both. Cells transfected with empty plasmid were used as control. n = 3; Error bars = SD; **p < 0.01. (**B**) qPCR analysis of the relative TGFB1 expression in C42B and 22Rv1 cells upon knockdown of AR, RARA, and RXRA expression alone or in combination. Cells transfected with scrambled siRNA (siScr) were used as a control. n = 3; Error bars = SD; *p < 0.05; **p < 0.01; ***p < 0.001. (**C**) A putative RARA-RXRA and AR binding motifs identified in the TGFB1 gene promoter using the Eukaryotic Promoter Database (https://epd.expasy.org/epd/). (**D**) LNCaP cells were treated with 5 ng/ml of TGFβ1 for 48 h, and MMP11 levels were analyzed by qPCR. n = 3; Error bars = SD; n.s. – non significant. (**E**) Analysis of the relative MMP11 and MMP26 gene expression in PCa and other cancer cell lines using DepMap portal (https://depmap.org/portal/); TPM – transcript per million. (**F**) qPCR analysis of the relative MMP11 expression in C4-2B cells upon ALDH1A1 and ALDH1A3 knockdown. Cells transfected with scrambled siRNA (siScr) were used as controls. n = 3; Error bars = SD; *p < 0.05; **p < 0.01. (**G**) qPCR analysis of the relative MMP11 expression in LNCaP and PC3 cells upon MMP11 knockdown. Cells transfected with scrambled siRNA (siScr) were used as controls. n ≥ 3; Error bars = SD; ***p < 0.001. (**H**) Plating efficiencies (PE, %) of LNCaP and PC3 cells after siRNA-mediated knockdown of MMP11. Cells transfected with scrambled (Scr) siRNA were used as controls. n = 3; Error bars = SD. There are no statistically significant differences in plating efficiency among the different conditions (n.s.).

Figure S3. (**A**) Relative cell radiosensitivity was analyzed by 2D radiobiological colony forming assay after siRNA-mediated knockdown of MMP11 in C4-2B and 22Rv1 cells. Cells transfected with scrambled (Scr) siRNA were used as controls. Error bars = SD; ***p<0.001. (**B**) Plating efficiencies (PE, %) of C4-2B and 22Rv1 cells after siRNA-mediated knockdown of MMP11. Cells transfected with scrambled (Scr) siRNA were used as controls. n = 3; Error bars = SD. There are no statistically

significant differences in plating efficiency among the different conditions (n.s.). (**C**) qPCR analysis of the relative MMP11 expression in C4-2B and 22Rv1 cells upon MMP11 knockdown. Cells transfected with scrambled siRNA (siScr) were used as controls. n = 3; Error bars = SD; **p < 0.001; ***p < 0.001. (**D**) Analysis of relative cell radiosensitivity in RM1(BM) cells stably transfected either with pLKO.1 puro vector constructs expressing shRNA against mouse Mmp11 (shMmp11#1 and shMmp11#2) or nonspecific control shRNA (shNS). Error bars = SD; ***p<0.001. (**E**) Plating efficiency analysis of (**D**). (**F**) qPCR analysis of the relative Mmp11 expression in RM1(BM) cells upon Mmp11 knockdown. Cells transfected with nonspecific shRNA (shNS) were used as controls. n ≥ 3; Error bars = SD; **p < 0.001; ***p < 0.001.

Figure S4. (**A**) Correlation of the MMP11 and (**B**) MMP26 gene expression levels with clinical attributes. Analysis was performed using cBioPortal for cancer genomics (https://www.cbioportal.org).

Figure S5. (**A**) The Kaplan-Meier analyses of the association of MMP11 expression and disease-free survival in a retrospective multicenter cohort including patients diagnosed with locally advanced PCa (Manchester dataset, Table 3). (**B**) Distribution of the relative gene expression levels in the TCGA PRAD dataset.

Figure S6. (**A**) The Kaplan-Meier analyses of the association of MMP11 plasma levels and PSA increase (>1 ng/ml above nadir) in patients with oligometastatic PCa treated with local ablative radiotherapy, n = 30. (**B**) A cutoff scan for the ROC analysis is shown in Figure 6D. (**C**) The MMP11 gene expression in tumor samples and normal tissues. The data are obtained using GEPIA 2. TCGA Study Abbreviations: ACC, Adrenocortical carcinoma; BLCA, Bladder Urothelial Carcinoma; LGG, Brain Lower Grade Glioma; BRCA, Breast invasive carcinoma; CESC, Cervical squamous cell carcinoma and endocervical adenocarcinoma; CHOL, Cholangiocarcinoma; COAD, Colon adenocarcinoma; ESCA, Esophageal carcinoma; GBM:Glioblastoma multiforme; HNSC, Head and Neck squamous cell carcinoma; KICH, Kidney Chromophobe; KIRC, Kidney renal clear cell carcinoma; KIRP, Kidney renal papillary cell carcinoma; LAML, acute myeloid leukemia; LIHC, Liver hepatocellular carcinoma; LUAD, Lung adenocarcinoma; LUSC, Lung squamous cell carcinoma; DLBC, Lymphoid Neoplasm Diffuse Large B-cell Lymphoma; OV, Ovarian serous cystadenocarcinoma; PAAD, Pancreatic adenocarcinoma; PCPG, Pheochromocytoma and Paraganglioma; PRAD, Prostate adenocarcinoma; READ, Rectum adenocarcinoma; SARC, Sarcoma; SKCM, Skin Cutaneous Melanoma; STAD, Stomach adenocarcinoma; TGCT, Testicular Germ Cell Tumors; THYM, Thymoma; THCA, Thyroid carcinoma; UCS, uterine carcinosarcoma; UCEC, Uterine Corpus Endometrial Carcinoma; TPM, transcripts per million. (**D**) Analysis of the MMP11 plasma levels in the NMRI (nu/nu) healthy and tumor-bearing mice.

Figure S7. Functional classification of the proteins identified by plasma proteomic profiling (**A**), associated pathways (**B**), and molecular functions according to PANTHER GO-slim (**C**). All analyses were performed using PANTHER18.0 classification system. (**D**) Characterization of 8 selected non-

immunoglobulin proteins regarding their comparative expression in high-risk (HR) vs. low-risk (LR) groups and correlation with time to PSA increase (20% above nadir); PCC: Pearson correlation coefficient.

Supplementary Figure 1

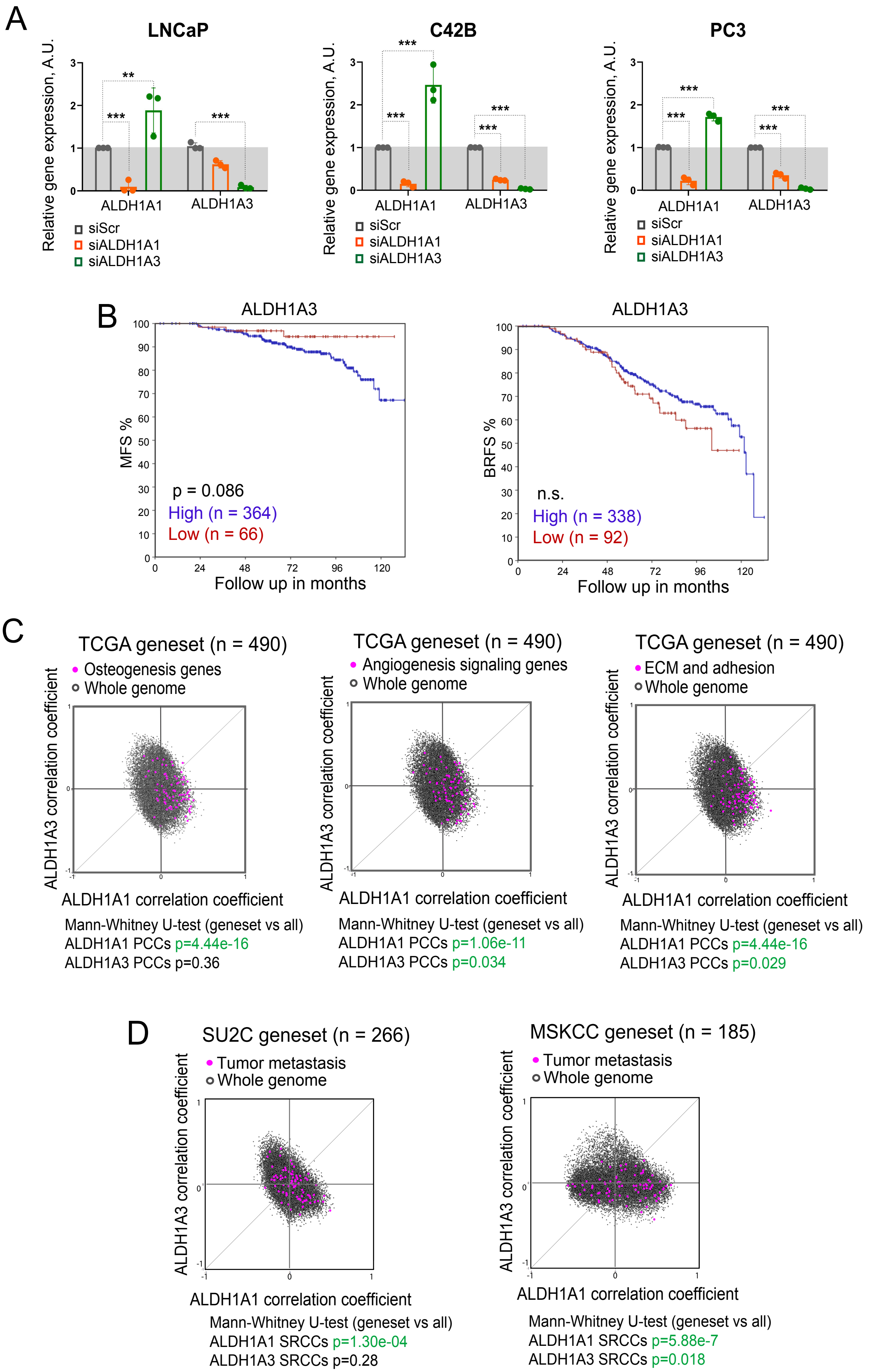

Supplementary Figure 2

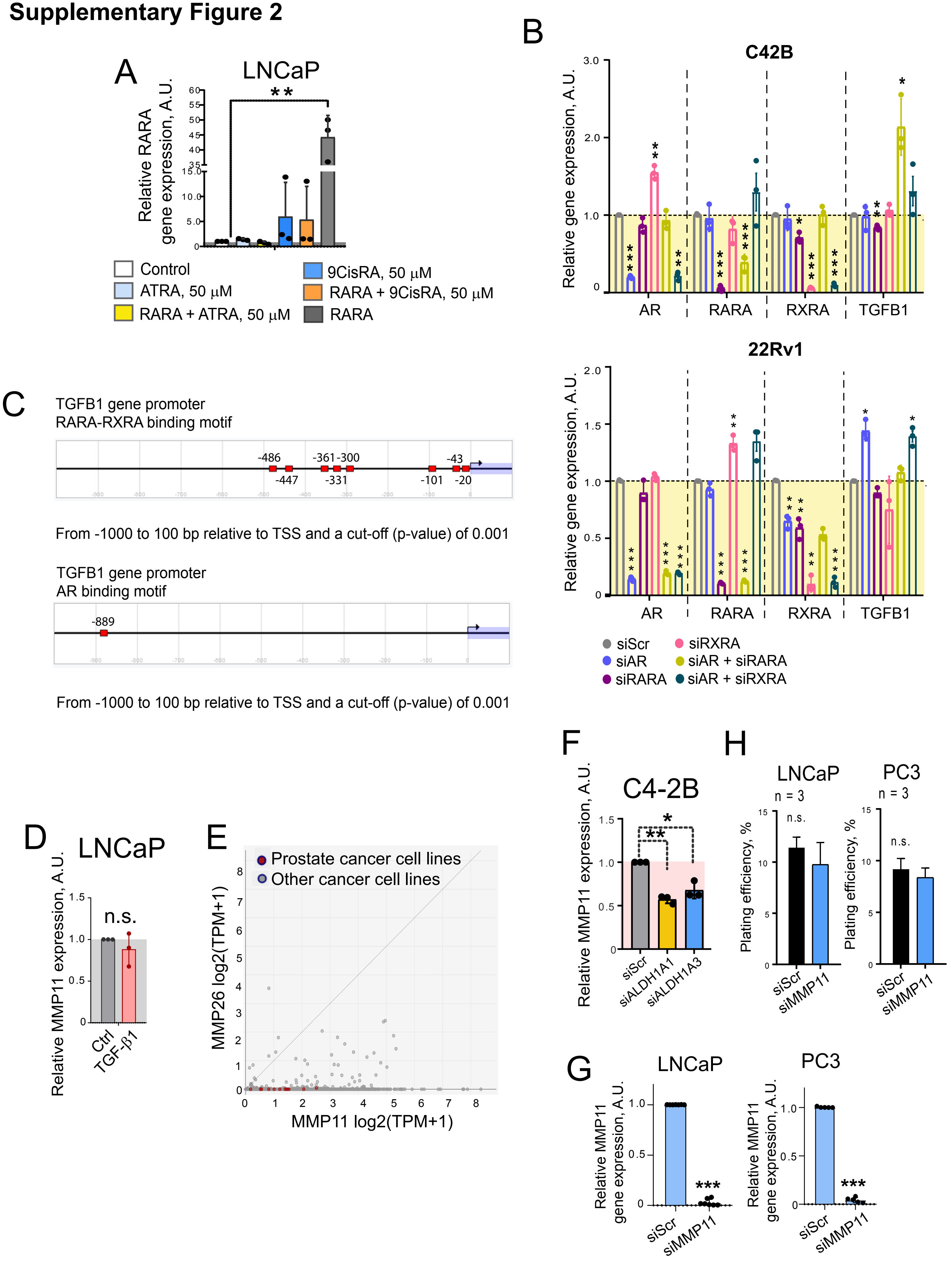

Supplementary Figure 3

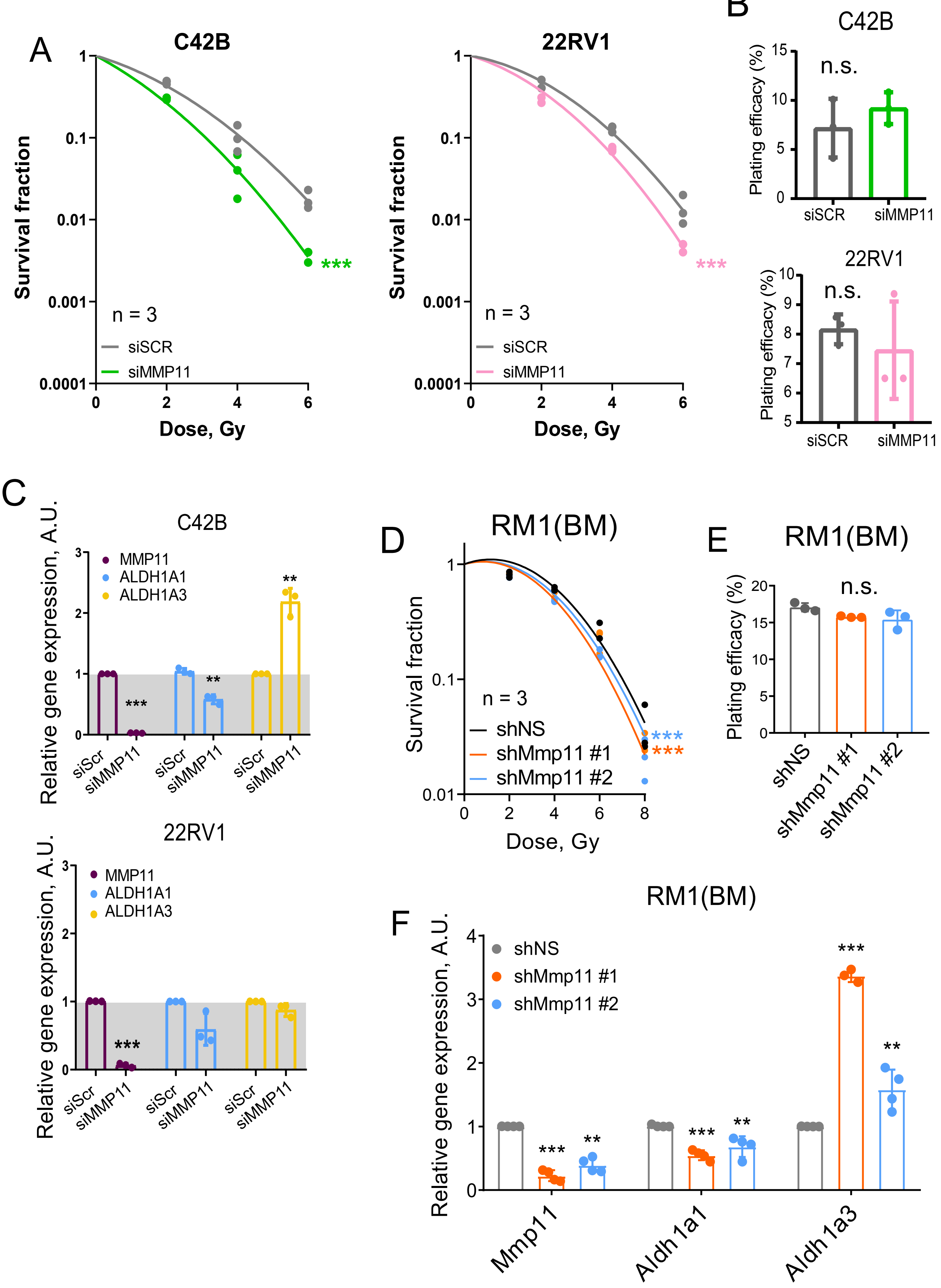

Supplementary Figure 4

A

TCGA, N = 498, MMP11

Clinical Attribute	Attribute Type	Statistical Test	p-Value	q-Value ▲
**Radical Prostatectomy Gleason Score for Prostate Cancer**	Patient	Chi-squared Test	**$< 10^{-10}$**	**$< 10^{-10}$**
**Gleason pattern primary**	Patient	Kruskal Wallis Test	**$< 10^{-10}$**	**$< 10^{-10}$**
**Fraction Genome Altered**	Sample	Kruskal Wallis Test	**$< 10^{-10}$**	**$< 10^{-10}$**
**American Joint Committee on Cancer Tumor Stage Code**	Patient	Chi-squared Test	**$< 10^{-10}$**	**1.77e-10**
**Person Neoplasm Status**	Patient	Chi-squared Test	**1.63e-7**	**2.252e-6**
**Mutation Count**	Sample	Kruskal Wallis Test	**2.076e-6**	**2.387e-5**
**Gleason pattern secondary**	Patient	Kruskal Wallis Test	**3.174e-6**	**3.129e-5**
**Positive Finding Lymph Node Hematoxylin and Eosin Staining Microscopy Count**	Patient	Kruskal Wallis Test	**8.836e-5**	**7.621e-4**

MSKCC, N = 150, MMP11

Clinical Attribute	Attribute Type	Statistical Test	p-Value	q-Value ▲
Radical Prostatectomy Gleason Score for Prostate Cancer	Sample	Chi-squared Test	5.218e-3	0.0587
Radical Prostatectomy Gleason Score for Prostate Cancer	Sample	Chi-squared Test	5.871e-3	0.0587
Neoplasm American Joint Committee on Cancer Clinical Primary Tumor T Stage	Patient	Chi-squared Test	0.0396	0.198
Sample Type	Sample	Chi-squared Test	0.0978	0.326
Sequenced	Sample	Chi-squared Test	0.180	0.399
Radical Prostatectomy Gleason Score for Prostate Cancer	Sample	Chi-squared Test	0.201	0.399
Mutation Count	Sample	Kruskal Wallis Test	0.212	0.399
Somatic Status	Sample	Chi-squared Test	0.220	0.399
Complete Data	Sample	Chi-squared Test	0.220	0.399
American Joint Committee on Cancer Tumor Stage Code	Patient	Chi-squared Test	0.341	0.569

DKFZ, N = 118, MMP11

Clinical Attribute	Attribute Type	Statistical Test	p-Value	q-Value ▲
**Radical Prostatectomy Gleason Score for Prostate Cancer**	Sample	Chi-squared Test	**4.841e-4**	**6.778e-3**
**TMB (nonsynonymous)**	Sample	Kruskal Wallis Test	**1.239e-3**	**8.670e-3**
**Mutation Count**	Sample	Kruskal Wallis Test	**2.627e-3**	**0.0105**
**ETS Status**	Sample	Chi-squared Test	**2.987e-3**	**0.0105**
**Time from Surgery to BCR/Last Follow Up**	Patient	Kruskal Wallis Test	**4.710e-3**	**0.0132**
**Preop PSA**	Patient	Kruskal Wallis Test	**0.0199**	**0.0465**
Stage	Patient	Chi-squared Test	0.0595	0.119
Median Purity	Sample	Kruskal Wallis Test	0.0703	0.123
Clonality	Sample	Chi-squared Test	0.0816	0.127
Diagnosis Age	Patient	Kruskal Wallis Test	0.0908	0.127
BCR Status	Patient	Chi-squared Test	0.138	0.176
Mono or Multifocal Status	Sample	Chi-squared Test	0.254	0.296
Number of Samples Per Patient	Patient	Chi-squared Test	0.331	0.356

B

TCGA, N = 498, MMP26

Clinical Attribute	Attribute Type	Statistical Test	p-Value	q-Value ▲
**Radical Prostatectomy Gleason Score for Prostate Cancer**	Patient	Chi-squared Test	**5.50e-10**	**3.80e-8**
**American Joint Committee on Cancer Tumor Stage Code**	Patient	Chi-squared Test	**3.53e-9**	**1.22e-7**
**Gleason pattern primary**	Patient	Kruskal Wallis Test	**2.07e-7**	**4.757e-6**
**Gleason pattern secondary**	Patient	Kruskal Wallis Test	**3.204e-5**	**5.528e-4**
**Sample Initial Weight**	Sample	Kruskal Wallis Test	**1.281e-4**	**1.768e-3**
**Specimen Second Longest Dimension**	Sample	Kruskal Wallis Test	**6.967e-4**	**6.604e-3**
**Patient Primary Tumor Site**	Patient	Chi-squared Test	**7.163e-4**	**6.604e-3**
**Did patient start adjuvant postoperative radiotherapy?**	Patient	Chi-squared Test	**7.953e-4**	**6.604e-3**
**Psa most recent results**	Patient	Kruskal Wallis Test	**9.493e-4**	**6.604e-3**

MSKCC, N = 150, MMP26

Clinical Attribute	Attribute Type	Statistical Test	p-Value	q-Value ▲
**Sample Class**	Sample	Chi-squared Test	**3.124e-4**	**6.247e-3**
**ERG Fusion GEX**	Sample	Chi-squared Test	**1.905e-3**	**0.0191**
ERG Fusion ACGH	Sample	Chi-squared Test	0.0143	0.0950
Fraction Genome Altered	Sample	Kruskal Wallis Test	0.0408	0.204
Radical Prostatectomy Gleason Score for Prostate Cancer	Sample	Chi-squared Test	0.0600	0.240
Somatic Status	Sample	Chi-squared Test	0.0901	0.255
Complete Data	Sample	Chi-squared Test	0.0901	0.255
Sample Type	Sample	Chi-squared Test	0.102	0.255
Radical Prostatectomy Gleason Score for Prostate Cancer	Sample	Chi-squared Test	0.219	0.390
TMB (nonsynonymous)	Sample	Kruskal Wallis Test	0.234	0.390
Sequenced	Sample	Chi-squared Test	0.330	0.473
Sequencing	Sample	Chi-squared Test	0.336	0.473
Neoplasm American Joint Committee on Cancer Clinical Primary Tumor T Stage	Patient	Chi-squared Test	0.371	0.473

DKFZ, N = 118, MMP26

Clinical Attribute	Attribute Type	Statistical Test	p-Value	q-Value ▲
**ETS Status**	Sample	Chi-squared Test	**5.115e-5**	**7.161e-4**
**Stage**	Patient	Chi-squared Test	**1.896e-3**	**0.0133**
TMB (nonsynonymous)	Sample	Kruskal Wallis Test	0.0459	0.162
Radical Prostatectomy Gleason Score for Prostate Cancer	Sample	Chi-squared Test	0.0476	0.162
Initial Treatment	Patient	Chi-squared Test	0.0690	0.162
Preop PSA	Patient	Kruskal Wallis Test	0.0696	0.162
Diagnosis Age	Patient	Kruskal Wallis Test	0.113	0.227
Mutation Count	Sample	Kruskal Wallis Test	0.138	0.242
Time from Surgery to BCR/Last Follow Up	Patient	Kruskal Wallis Test	0.171	0.266
Clonality	Sample	Chi-squared Test	0.286	0.401
BCR Status	Patient	Chi-squared Test	0.431	0.548
Number of Samples Per Patient	Patient	Chi-squared Test	0.661	0.771
Median Purity	Sample	Kruskal Wallis Test	0.803	0.865
Mono or Multifocal Status	Sample	Chi-squared Test	0.900	0.900

Supplementary Figure 5

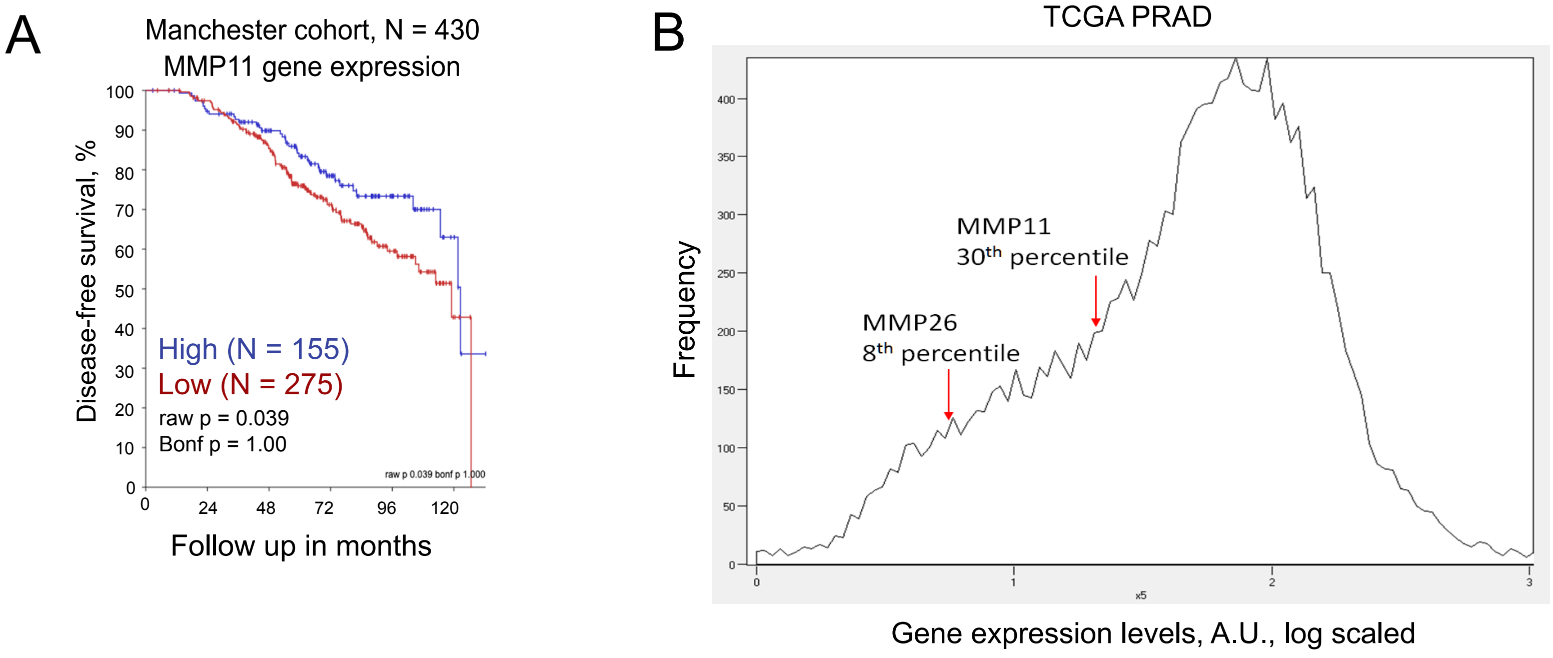

Supplementary Figure 6

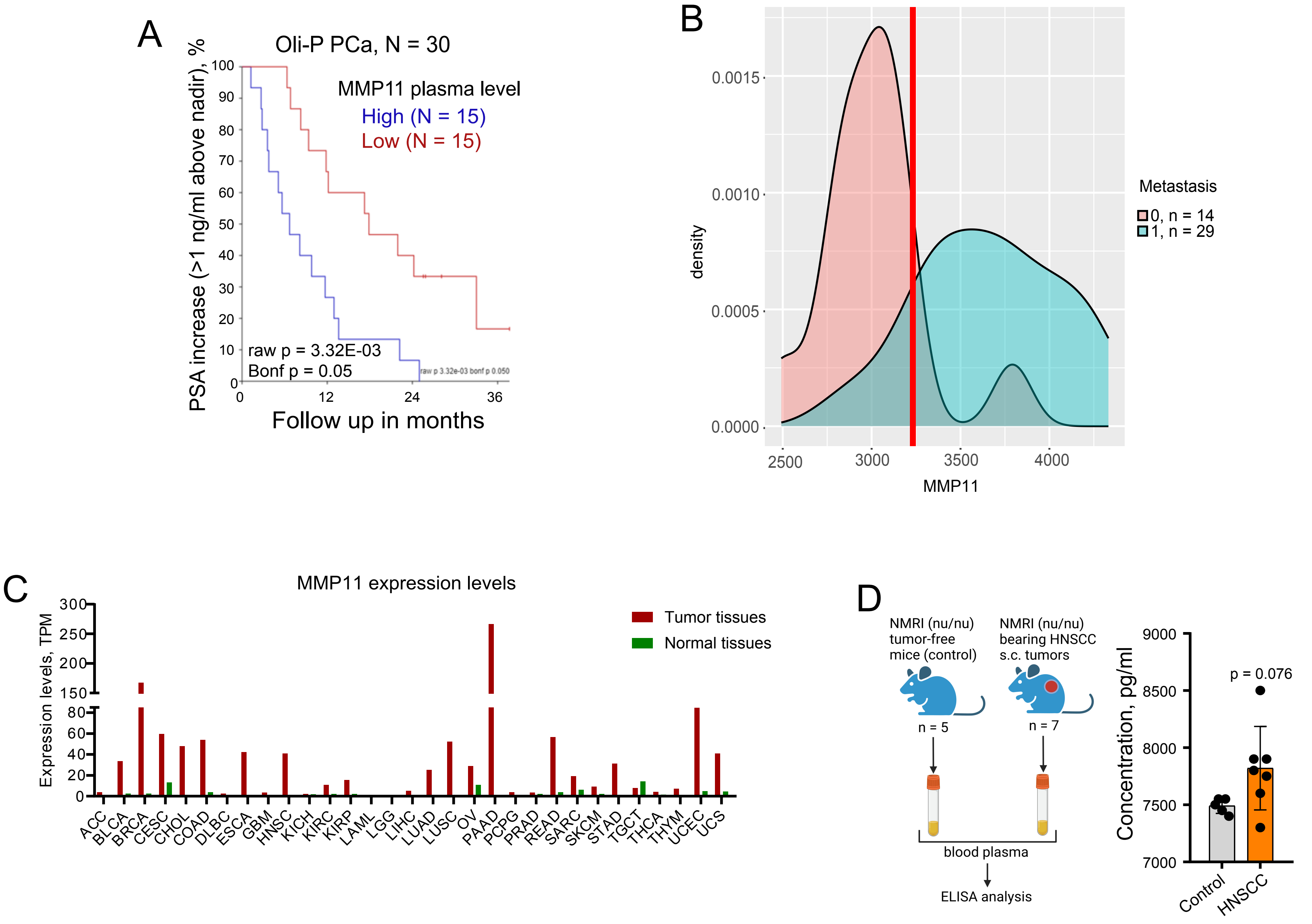

Supplementary Figure 7

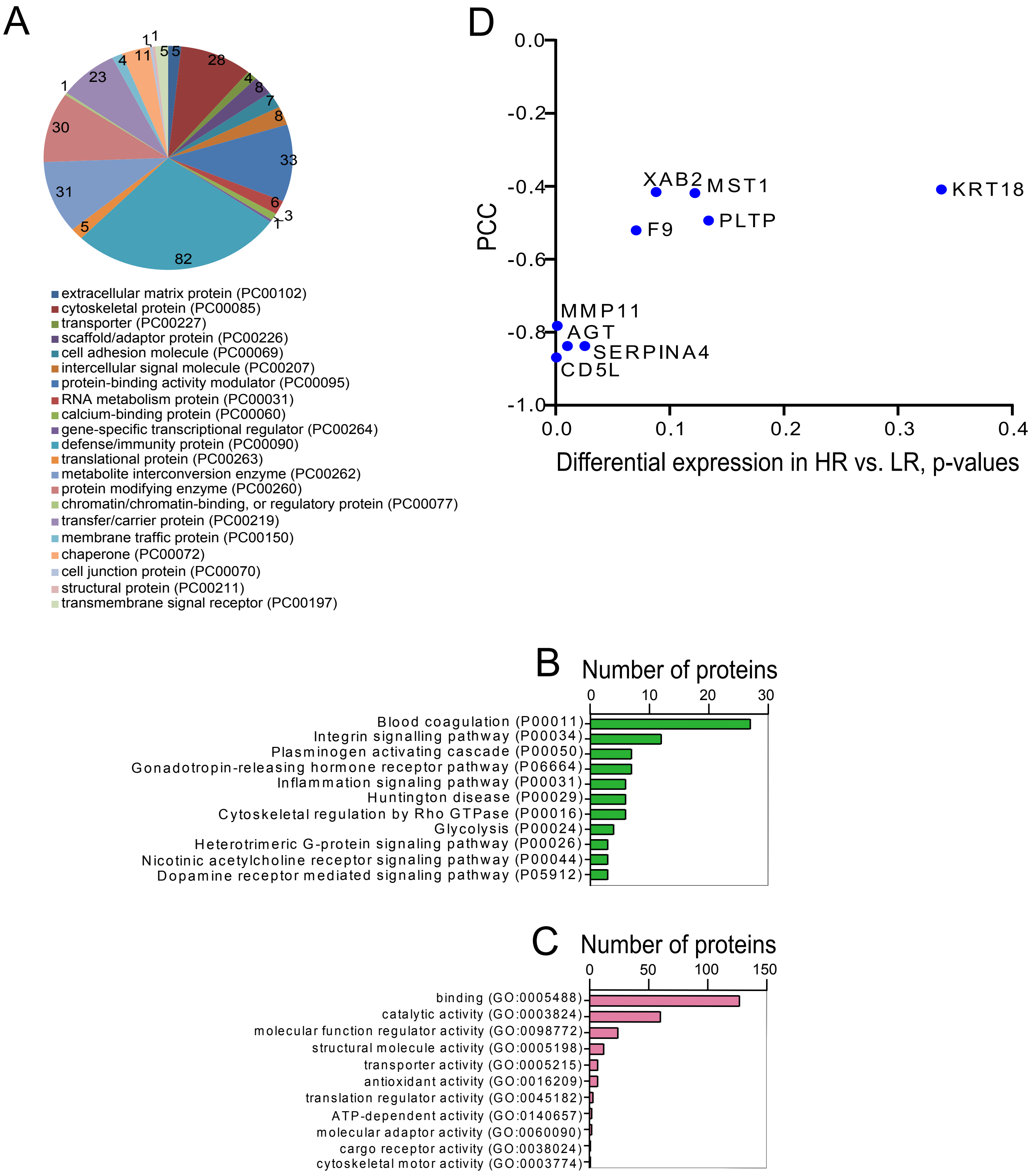

Supplement: Supplementary file 1 — Supplementary Material 1. [file 13046_2025_3299_MOESM1_ESM.zip › 300125 Supplementary information_ESM.pdf]
